# A COVID-Positive 52-Year-Old Man Presented With Venous Thromboembolism and Disseminated Intravascular Coagulation Following Johnson & Johnson Vaccination: A Case-Study

**DOI:** 10.7759/cureus.16383

**Published:** 2021-07-14

**Authors:** Omar Shazley, Moudar Alshazley

**Affiliations:** 1 Basic Sciences, Saint James School of Medicine, Kingstown, VCT; 2 Emergency Medicine, Internal Medicine, Santa Rosa Medical Center, Pensacola, USA

**Keywords:** saddle pulmonary embolism, venous thromboembolism (vte), johnson and johnson vaccine, coronavirus covid-19, thrombotic thrombocytopenic thrombocytopenia, disseminated intravascular coagulation (dic), deep vein thrombosis (dvt)

## Abstract

The coronavirus disease 2019 (COVID-19) is caused by the severe acute respiratory syndrome coronavirus type 2 (SARS-CoV-2). Infection by the SARS-CoV-2 increases the risk for systematic multi-organ complications and venous, arterial thromboembolism. The need for an effective vaccine to combat the pandemic prompted the Centers for Disease Control and Prevention (CDC) and Food and Drug Administration (FDA) to approve a nationwide distribution of the Ad26.COV2.S vaccine manufactured by Johnson & Johnson (J&J). The use of the vaccine was halted after reported cases of cerebral venous sinus thrombosis (CVST) and thrombocytopenia among recipients. Researchers have postulated these rare occurrences as potentially immune-triggered responses associated with complement-mediated thrombotic microangiopathy (TMA). Thrombotic complications and thrombocytopenia increase the risk for blood clot growth due to the inflammation of immune complexes by pro-thrombotic activation of anti-platelet antibodies.

A 52-year-old man presented to the intensive care unit (ICU) with severe dyspnea. He required bilevel positive airway pressure (BiPAP) for supplemental oxygen therapy. Endotracheal intubation was performed due to his worsened respiratory deterioration. Lab results suggested respiratory failure due to decreased partial pressure of oxygen (pO_2_) and increased partial pressure of carbon dioxide (pCO_2_). Findings of elevated D‐dimer levels with decreased fibrinogen and thrombocytopenia with prolonged prothrombin clotting time were consistent for disseminated intravascular coagulation (DIC). Chest radiography displayed moderate to heavy bilateral airspace consolidations, consistent with multifocal pneumonia suspicious for COVID-19. A computed tomography angiogram (CTA) revealed a mildly enlarged right ventricle and interventricular septum consistent for right heart strain due to a saddle pulmonary embolism (PE) that extended into the main pulmonary lobar segmental arteries bilaterally. The patient was transferred to a higher-level (tertiary) care for radiology intervention to remove the pulmonary embolism found on his lungs.

This patient presented with severe dyspnea secondary to massive PE and deep venous thrombosis (DVT) due to SARS-CoV2 infection following the administration of the J&J vaccine. Bilateral thrombus opacities and pulmonary emboli are consistent among COVID-19 patients by intravascular coagulation with increased prothrombin time and D-dimer concentration with a low platelet count. Adverse emboli growths with increased D-dimer and thrombocytopenia strikes a similarity in recipients of the AstraZeneca vaccine due to vaccine-induced immune thrombotic thrombocytopenia (VITT). Administrative use of the J&J vaccine resumed in May 2021. The FDA's reassurance stemmed from their conclusive findings that the vaccine's benefits far outweigh these rare developments, which account for less than 0.01% of the total recipient population. Nevertheless, a further detailed analysis must be conducted on the adverse thrombotic manifestations following adenoviral-based COVID-19 vaccines (J&J, AstraZeneca) compared to mRNA-based vaccines (Moderna, Pfizer) to assess causality with higher specificity.

## Introduction

As of July 2021, the pandemic has infected more than 183 million people and has resulted in more than 3.9 million deaths worldwide [1]. It is well documented that the most common cause of death by COVID-19 is an acute hypoxic respiratory failure from acute respiratory distress syndrome (ARDS). Complications of thromboembolism and prothrombotic coagulation have recently emerged in critical COVID-19 patients [2]. Pulmonary embolism (PE) statistically accounts for 63% of thromboembolic complications in COVID-19 [3].

In response to increased nationwide hospitalizations for COVID-19, the Food and Drug Administration (FDA), on February 27, 2021, issued an Emergency Use Authorization (EUA) for the Ad26.COV2.S vaccine manufactured by Johnson & Johnson (J&J) [4]. It uses a replication-incompetent human adenoviral type 26 vector platform when administered as a single intramuscular dose [5]. Approximately 6.8 million dosages of the J&J vaccine were recorded by April 21, 2021, until administrations were halted when six cases of cerebral venous sinus thrombosis (CVST) with thrombocytopenia (platelet count <150,000/μL of blood) were reported among vaccine recipients [6]. The Vaccine Adverse Event Reporting System (VAERS) received 15 additional reports of thrombosis with thrombocytopenia syndrome (TTS). The decrease in platelet count found in TTS strikes a similarity to heparin-induced thrombocytopenia (HIT), in which platelet-activated antibodies develop in the absence of exposure to heparin [7].

We present a case of venous thromboembolism (VTE) by severe growths of PE and deep vein thrombosis (DVT) with disseminated intravascular coagulation (DIC) in a COVID-19-positive patient following the administration of the J&J vaccine.

## Case presentation

A 52-year-old man with a history of hypertension, hyperlipidemia, obesity, gastroesophageal reflux disease (GERD), and non-insulin-dependent diabetes mellitus (NIDDM) presented to the ICU with a 10-day onset of dyspnea and a dry cough. He was a former cigarette smoker with a 10-year history of 15 packs per day but had stopped for the past two years. He stood 180.3 cm tall and weighed 151.5 kg for a body mass index (BMI) of 46.6. EMS reported the patient’s O_2_ saturation at 57% room air and immediately placed him on BiPap for supplemental oxygen therapy upon arrival. The patient reported his shortness of breath had worsened in the last 24 hours. Aggravating factors included exertion and an attempt to speak. A positive PCR test confirmed the patient’s diagnosis for COVID-19 on April 10, 2021, approximately eight days after he was exposed by a co-worker on April 2, 2021 (Table 1). These sequences of events occurred following his administration of the J&J vaccine on March 31, 2021 (Table 1). His review of systems was remarkable for exacerbating chest pain and a dry cough with a worsened shortness of breath. He had a family history significant for hypertension. He denies any tobacco, alcohol, or drug abuse. He had no known drug allergies (NKDA). His regularly prescribed medications included Atorvastatin, Metoprolol, and Pantoprazole.

**Table 1:** Timeline of the patient's vaccine administration and COVID-19 infection before arrival.

Date	Sequence outline
March 3, 2021	The patient received the J&J vaccine.
April 2, 2021	Exposure to COVID-19 by a co-worker.
April 10, 2021	Positive PCR test confirmed diagnosis for COVID-19.
April 21, 2021	Admittance to the ICU for worsening dyspnea and cough.

An electrocardiogram (ECG) obtained en route reported regular rhythm with sinus tachycardia with no signs of ectopy. The PR, QRS, and T-waves were normal with no changes to his ST-wave. On arrival, he displayed severe respiratory deterioration with dyspnea and hyperventilation with measured pulse oximetry of room air at 67%. Cardiac defibrillation was successfully performed by endotracheal tube (ET) intubation; 40 mg of propofol, 4 mg versed, and 50 mg of rocuronium were administered to sedate the patient.

Physical examination was notable for a heart rate of 136 beats per minute, a respiration rate of 40 breaths per minute, and hypertension (174 mmHg/104 mmHg). The cardiovascular examination was significant for rhythm sinus tachycardia with S1 and S2 heart tones present. For his pulmonary examination, he required mechanical ventilation at a rate of 15 liters per minute, 100% BiPap, and 5 cmH_2_O for positive end-expiratory pressure (PEEP). Bilateral crackle sounds were heard, along with an observed asymmetrical chest mount and intercostal retractions. The use of his accessory muscles was noted.

**Figure 1:**
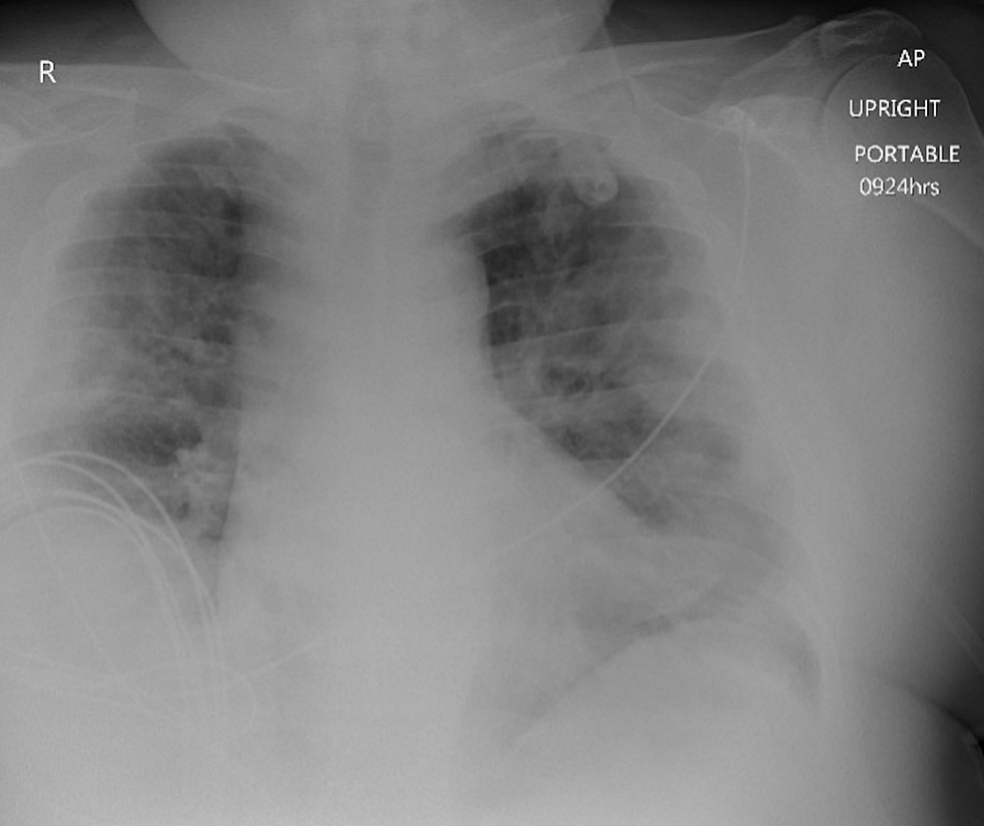
Patient’s initial emergency department chest radiograph. Bilateral airspace consolidations with no acute osseous abnormalities are shown, a consistency found with acute respiratory distress syndrome.

**Figure 2:**
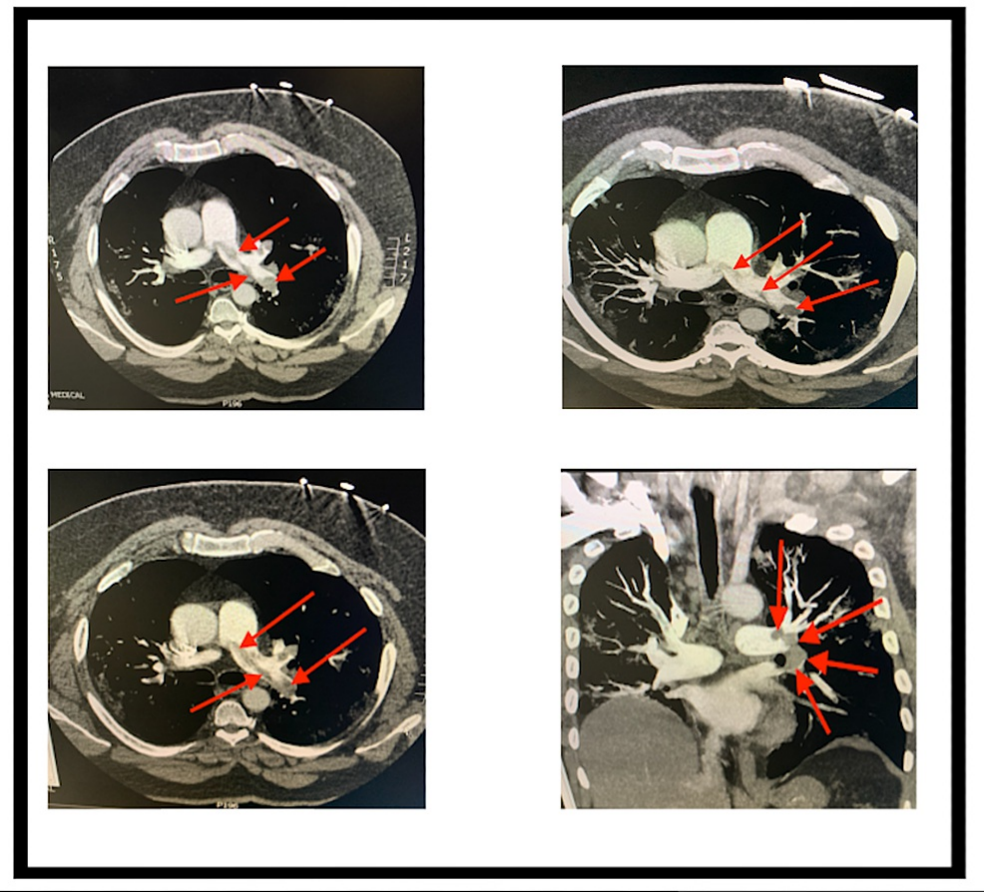
CT pulmonary angiography with coronal, sagittal, and maximum intensity projection (MIP) reconstructions. The images show thrombus crossing the bifurcation extending into the right upper, middle, and lower lobe segmental branches with a greater thrombus burden on the left pulmonary arteries (arrows).

**Figure 3:**
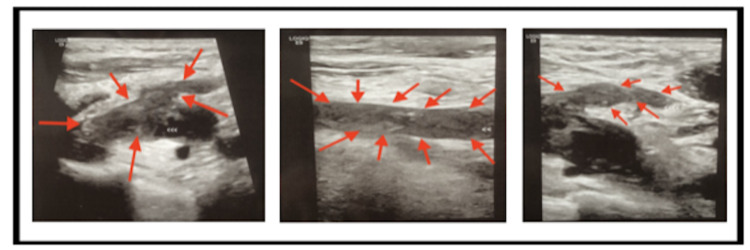
Ultrasound of bilateral lower extremities by Grayscale Doppler with color flow spectral broadening. These demonstrate extensive bilateral deep venous thrombosis.

Given the report of a positive COVID-19 diagnosis with severe dyspnea and hyperventilation, there was high suspicion for SARS-associated pneumonia as the cause for his ARDS. He received an RNA nose swab and was started on a combination of Remdesivir, Rocephin, Zithromax, and Decadron (6 mg IV q 12 hours). Vaportherm was provided for additional oxygenation along with normal saline 150 cc/hour for sepsis. He was emergently taken to radiology for suspected pulmonary masses. Chest X-rays revealed bilateral airspace consolidations with no acute osseous abnormalities to confirm lateral airspace disease (Figure 1). These consolidations would likely be a result of an infection/inflammation due to sepsis or viral pneumonia. The catheterization lab revealed an elevated filling pressure on the right side of his heart with a right atrial pressure of 21 mmHg and a pulmonary capillary wedge pressure of 14 mmHg. A computed tomography (CT) angiogram revealed a thrombus crossing the bifurcation that extended into the right upper, middle, and lower lobe segmental branches with a greater thrombus burden on the left pulmonary arteries and the right ventricle (Figure 2). Minimal atheromatous plaques were found with no dissection or thoracic aortic aneurysm. Bilateral ground-glass consolidations with interstitial thickening were discovered in the lungs with an increased right to left heart ratio suggesting an underlying right heart strain to a certain degree. Upper and lower extremity Doppler ultrasounds showed occlusive and non-occlusive thrombus bilaterally on the patient’s lower extremity veins (Figure 3).

Laboratory analysis displayed in Table 2 was conducted upon the patient’s arrival. It showed increased concentrations of glucose (164 mg/dL), alanine (82 U/liter), and aspartate aminotransferase (43 U/liter) with a low platelet count of 100,000 μL. Low levels of fibrinogen at 132 mg/dl were measured. Elevated prothrombin time (PT) and partial-thromboplastin (PTT) were measured at 16.4 and 37 seconds, respectively. Elevated acidity levels were observed for the patient with increased lactic acid levels from 9.0 mmol/L to 10.3 mmol/L (Table 2) and a decreased pH from 7.455 to 7.255 (Table 3). The patient’s arterial blood gas values in Table 3 revealed a low partial pressure of oxygen (pO_2_) of 58.3 mmHg and increased partial pressure of carbon dioxide (pCO_2_) of 40.2 mmHg. These blood gas values put the patient at a higher risk for respiratory failure with a decreased PaO_2_/FiO_2_ ratio of 58.3 mmHg/100%. This ratio meets the threshold feature for increased severity of ARDS [8]. Severe hypoxia with a PaO_2_/FiO_2_ ratio less than 250 is associated with community-acquired pneumonia (CAP) [9]. He had an abnormally elevated D-dimer level at 3.6 μg/mL (Table 2).

**Table 2:** Laboratory characteristics of the patient at the time of arrival and transfer.

Laboratory analysis	9.00 am	4.00 pm	Reference value
Hemoglobin (g/dl)	11.1	10.9	12.0–16.0
Platelet count (per mm^3^)	100,000	72,000	150,000–350,000
Leukocytes (per mm^3^)	17,100	18,500	4,000–10,000
Partial thromboplastin time (sec)	37	45	<35
Thrombin time (sec)	16.4	17.0	12-14
D-dimer (μg/mL)	3.6	4.0	<0.5
Fibrinogen (mg/dl)	132	95	200–400
Aspartate aminotransferase (U/liter)	43	60	<35
Alanine aminotransferase (U/liter)	82	92	<35
Glucose (mg/dL)	164	NA	70–100
Lactic acid (mmol/L)	9.0	10.3	0.5–2.2
Neutrophil (%)	80.6%	NA	45–75%
Absolute neutrophil (per mm^3^)	14,100	NA	1,500–8,000
Lymphocyte (%)	10.3%	9.8%	15–45%

**Table 3:** Arterial blood gas laboratory characteristics of the patient at arrival and transfer.

Laboratory analysis	9:00 a.m.	4:00 p.m.	Reference value
pH	7.45	7.25	7.35–7.45
pCO_2_ (per mmHg)	40.2	67.6	35–45
pO_2_ (per mmHg)	58.3	50.8	80–100
Base excess (mmol/L)	4	1	−2 to +2
HCO_3_ (mmol/L)	28.2	30.0	22–26
O_2_ saturation (%)	90.0%	76.1%	93–100%
FiO2 (%)	100%	100%	100%
Liter flow (per L/min)	15	NA	NA
PEEP (cmH_2_O)	NA	5	>5

The patient continued hyperventilating with a decline in oxygen saturation from 90% to 76.1% (Table 3). He was deemed critical but in stable condition within the capability of the ICU. The attending physician explained to the patient and his family the status of his continued respiratory failure by the findings of the DVT and PE. The patient was transferred upon recommendations made by his attending physician and the intensivist to a nearby healthcare facility for higher-level (tertiary) care for further management. He underwent radiological intervention two days later to remove the clots from his lungs. The patient, unfortunately, continued to experience worsened dyspnea with no significant improvement, which resulted in his expiration approximately one month following his initial admission.

## Discussion

​​This patient presented with severe dyspnea secondary to massive PE and DVT due to an infection by the SARS-CoV2 following the administration of the J&J vaccine. With current studies suggesting as many as 20-25% of admitted COVID-19 patients have presented with thromboembolism, recent reports of the syndrome termed VITT have resulted in findings of CVST and thrombocytopenia among recipients of the J&J vaccine [10,11].

Immune-triggered responses associated with complement-mediated thrombotic microangiopathy (TMA) are common among COVID-19 patients [12]. As our immune system generates formulated host defenses through random reassortment, the presence of an eluding mutated microorganism such as the SARS-CoV2 causes the body to counteract with the activation of local immune cells through the secretion of various cytokines such as IL-6 [12]. Such a lengthy, broadened countermeasure can become destructive by triggering extensive coagulative tissue damage [12]. Prolonging this dysregulated immune response would lead to widespread inflammation. The apparent enlargement of the patient’s pulmonary trunk is evident of the relatively large saddle PE that extended into the main pulmonary, lobar, and segmental arteries bilaterally to occlude the lower lobe pulmonary artery (Figure 2). Such an abnormality with a mildly enlarged right ventricle and interventricular septum leads to the prognosis for right heart strain. Bilateral thrombus opacities (Figure 1) present within COVID-19 patients might progress over a short time to more obvious pneumonia with respiratory failure and features of classic ARDS [12]. Extension of the patient’s large saddle PE contributed to his hypoxemia due to the SARS-CoV-2 infection. Similar to cardiotropic viruses, the SARS-CoV-2 attacks cardiac cells to lower the expression of ACE2 receptors that play a role in increasing blood pressure and inflammation [13]. Damage to the blood vessels and tissues by viral infection to the heart muscle warrants pro-inflammatory cytokine release due to coagulation [13]. The patient’s elevated D-dimer and decreased fibrinogen corresponds to the findings in a retrospective study conducted by Tang et al. where 183 COVID-19 patients in China had significantly higher concentrations of D‐dimer and fibrin degradation products [14]. Similar to this study’s laboratory findings, longer prothrombin time and activated partial thromboplastin time with low platelet counts were found among survivors consistent with classic DIC in the setting of SARS-CoV-2-induced sepsis [14].

Clinical findings of VTE and PE with thrombocytopenia and elevated D-dimer (Table 2) strike a similarity to those vaccinated in Europe with the AstraZeneca (ChAdOx1 nCoV-19) vaccine. The hallmarks of these cases included the formation of blood clots in the brain termed CVST and thrombocytopenia 4-20 days following vaccination [15]. The timeline of the patient’s vaccination (March 31, 2021) and positive COVID-19 test (4/10) follow this time frame by his arrival 21 days (4/21) after his initial vaccination. While his laboratory findings of low platelet count with elevated D-dimer and prolonged clotting prothrombin time are clinically consistent for VITT, no findings of CVST were made as he denied classic symptoms such as headaches, vision changes, faints, or confusion. 

This case study showcases a unique abnormality in regards to the possible diagnosis for VITT. Without prior heparin exposure, VITT displays findings of acute thrombotic complications and severe thrombocytopenia following administration of the J&J vaccine. A single dose of the adenoviral-associated vaccine prompts the pro-thrombotic activation of anti-PF4 antibodies through IgG-FcγR interactions and FcR-mediated engagement of immune effector cells [16]. C3 activation due to the anti-PF4 complex results in the downstream generation of potent pro-inflammatory effectors that can potentiate inflammation [16]. Deregulated complement C3 responses with circulating anti-PF4 immune complexes ignite complement activation of inflammatory properties of monocytes and neutrophils through complement receptors [16]. This development postulates that the role complement may have on vaccine-related adverse reactions. The activation has been implicated as a cause of thromboinflammation of immune complexes in autoimmune pathologies, increasing the risk for blood clots [16].

Findings of thrombosis within this male patient is a rare occurrence following the administration of the J&J vaccine, where the majority of recent cases of thrombosis with thrombocytopenia (TTS) have primarily affected women aged 18-49 years [7]. What is unique and informative about this case is the similarity in thrombotic developments recently found in female recipients of the AstraZeneca vaccine in Europe. While further research is needed to provide an adequate explanation for the endured manifestation of COVID-19 despite having received the J&J vaccine, we believe the patient’s obesity was a significant factor. Obesity increases the risk for thromboembolic growth due to the abnormal secretion of pro-inflammatory cytokines [17]. The increased concentration of adipose tissue enables the pathogenicity of COVID-19 by increasing pro-inflammatory response to various viral infection types [18]. At the age of 52 with a BMI of 46.6, the patient shares similarities with the recent findings in studies that have highlighted obesity as a risk factor for COVID-19 hospitalization in patients younger than 60 years of age where those who are young and severely obese with a BMI ≥ 40 are five times more likely to die [19].

Limitation

The patient’s medical findings at the hospital he was transferred to were not disclosed in this case study. Autopsy findings of the patient’s lungs were not obtained at the time of writing.

## Conclusions

This case study presented the adverse growths of DVT and PE possibly induced by the J&J vaccine. With the rarity of these developments accounting for less than 0.01% of the total vaccinated population, the CDC and FDA lifted the recommended pause of the J&J vaccine effectively on April 23, 2021. After a thorough safety review, both agencies concluded that the vaccine’s benefit to minimize SARS-CoV2 infection far outweighed the risk of developing any adverse effects. In the presence of acute thrombocytopenia/thrombosis, alternative HIT-compatible anticoagulants should be used until HIT is ruled out as the cause of these manifestations. Further analysis on the comparative manifestations of thromboembolism induced by adenoviral (J&J, AstraZeneca) and mRNA-based vaccines (Moderna, Pfizer) with patient characteristics (e.g., comorbidities) must be conducted to better assess causality with higher specificity.
